# Ferroptosis in Rheumatoid Arthritis: A Potential Therapeutic Strategy

**DOI:** 10.3389/fimmu.2022.779585

**Published:** 2022-02-02

**Authors:** Ting Zhao, Qi Yang, Yujiang Xi, Zhaohu Xie, Jiayan Shen, Zhenmin Li, Zhaofu Li, Dongdong Qin

**Affiliations:** School of Basic Medical Sciences, Yunnan University of Chinese Medicine, Kunming, China

**Keywords:** ferroptosis, rheumatoid arthritis, lipid peroxidation, reactive oxygen species, therapeutic strategy

## Abstract

Ferroptosis is one of the newly discovered forms of cell-regulated death characterized by iron-dependent lipid peroxidation. Extensive research has focused on the roles of ferroptosis in tumors, blood diseases, and neurological diseases. Some recent findings have indicated that ferroptosis may also be related to the occurrence and development of inflammatory arthritis. Ferroptosis may be a potential therapeutic target, and few studies *in vitro* and animal models have shown implications in the pathogenesis of inflammatory arthritis. This mini review discussed the common features between ferroptosis and the pathogenesis of rheumatoid arthritis (RA), and evaluated therapeutic applications of ferroptosis regulators in preclinical and clinical research. Some critical issues worth paying attention to were also raised to guide future research efforts.

## Introduction

Rheumatoid arthritis (RA) is the most specific systemic immune system disease among autoimmune diseases (1), invading many joints, such as knee and elbow joints. Its main clinical manifestations are joint swelling and stiffness in the morning. RA has an incidence of 0.5% to 1%, with an apparent reduction from north to south (in the northern hemisphere) and from urban to rural areas (2). Some Native American populations have a very high prevalence. The incidence of RA is high in 30-50 years of age, and the incidence in women is about three times that of men. RA may be related to various cell types and cytokines (3), and the origin of its pathology is autoantibodies (4, 5). RA is characterized by infiltration of macrophages and lymphocytes, the proliferation of synovial fibroblasts, joint inflammation, progressive cartilage destruction, and bone erosion, as well as degenerative manifestations (6).

Disease-modifying antirheumatic drugs (DMARDs) are conventional drugs in the treatment of RA. Depending on the symptom severity, RA can be treated with a single drug or a combination of 2, 3, or 4 drugs (7, 8). When the disease is refractory, some biologic DMARDs are recommended. The use rate of biological agents in the treatment of RA in North America has been as high as 50.7% (9). However, the clinical efficacy rate is inconsistent, ranging from 50% to 70% (10). In clinical practice, we should make changes in the therapeutic strategy when the arthritis is resistant to initial therapy. Hence, there is an urgent need to develop drugs with new targets or new mechanisms of action to meet the clinical needs of patients.

In RA, mature B cells and dendritic cells present antigens to T cells, leading to T cell activation. Different immune cells secrete unique cytokines and jointly stimulate the expression of cytokine TRANCE/receptor activator of nuclear factor-kappa B ligand (RANKL), which is necessary for osteoclast differentiation. B-T cell interaction leads to the activation of plasma cells responsible for producing and secreting autoantibodies. Autoantibodies, cytokines, and RANKL stimulate osteoclasts to cause bone resorption and induce cartilage damage driven by chondrocytes. In addition, compared with activated B cells, transitional B cells can inhibit the formation of osteoclasts in an immunomodulatory manner by providing IL-10. It is reported that ferroptosis plays a vital role in the occurrence and development of many diseases such as Parkinson’s disease, ischemia-reperfusion injury, and tumors (11, 12). Recent studies have shown that ferroptosis plays a critical regulatory role in autoimmune and inflammatory diseases (13, 14). New strategies for targeting ferroptosis are to regulate the immune response homeostasis, and in some cases, the reactions can influence each other. Studies have found that ZIP14, a ferroptosis-related metal transporter, may play a regulatory role in the immune system (15). Early studies have confirmed that glutathione peroxidase (GPX) activity in polymorphonuclear leucocytes of RA patients with high persistent disease activity is reduced (16). Luo et al. (17) found that RSL3, a ferroptosis activator, can induce ferroptosis in synovial cells and aggravate synovitis. Transferrin receptor 1 (TFR1) and nuclear receptor coactivator 4 (NCOA4) were upregulated, but system Xc- (an amino acid transporter mediating the exchange of extracellular cystine and intracellular glutamate) and GSH-glutathione peroxidase 4 (GPX4), as well as nuclear factor erythroid 2-related factor 2 (Nrf2, a transcriptional factor that induces antioxidative and cytoprotective responses), were downregulated by RSL3 treatment. Herein, ferroptosis may be a potential therapeutic target for inflammatory arthritis in the future.

This mini review discussed the common features between ferroptosis and the pathogenesis of RA, and evaluated therapeutic applications of ferroptosis regulators in preclinical and clinical research. Some critical issues worth paying attention to were also raised to guide future research efforts.

## Ferroptosis in Cell Death

Cell death is a sophisticated process, and its mechanisms have traditionally been divided into two types, programmed cell death (PCD) mechanisms that require energy, and necrotic cell death mechanisms that do not (18). In addition, necrotic cell death typically causes a strong immune response, whereas PCD does not (19, 20). In 2012, Dixon et al. (21) discovered a unique iron-dependent form of nonapoptotic cell death when studying the mechanism of the small molecule compound (named erastin) against RAS mutant tumors, which was called ferroptosis. It is significantly different from other death patterns in morphology, biochemistry, and genetics (22). It is characterized by the accumulation of lethal reactive oxygen species (ROS) arised from the reaction between iron and lipid peroxides, which are themselves generated by the oxidation of polyunsaturated fatty acids (PUFAs)-containing phospholipids (PUFA-PLs) (23). PUFAs are essential for ferroptosis due to their sensitivity to lipid peroxidation (24). Free PUFAs are involved in ferroptosis after they are esterified into PUFA-PL and PUFA-PLOOH. Ferroptosis does not have morphological characteristics of apoptosis, such as cell shrinkage, chromatin agglutination, formation of apoptotic bodies, disintegration of cytoskeleton, and other phenomena. However, it can be observed that the volume of mitochondria decreases and the membrane’s density increases (25), which are not observed in apoptosis. At the same time, along with mitochondrial morphology alterations accompanying ferroptosis, a common morphological feature is cell ballooning/blistering followed by plasma membrane rupture (26). In terms of biochemical characteristics, ferroptosis is mainly triggered by glutathione (GSH) depletion and glutathione peroxidase 4 (GPX4) inactivation. It is mainly related to lipid peroxidation metabolism and intracellular iron balance, and several genes are involved in the regulation of ferroptosis. Ferroptosis inducers mainly include erastin, FINO2, and RSL3. Ferroptosis inhibitors mainly include liproxstatin-1, iron chelator, and ferrostatin-1. Liproxstatin-1 free radicals, which were formed by removing lipid peroxides from liproxstatin-1, can be reduced by other antioxidants (such as ubiquinone). Zika et al. found that liproxstatin-1 may reduce the accumulation of intracellular toxic lipid ROS, thereby inhibit the occurrence of cell ferroptosis (27). At present, the understanding of ferroptosis is not comprehensive enough, and its mechanism is still in the exploratory stage.

## The Potential Role of Ferroptosis in RA

Ferroptosis might play a role in the onset of RA and may be used as a treatment option in the future. Recently, it has been found that RA and ferroptosis have similar characteristics, mainly in the following aspects.

## Iron Handling

**Figure 1:**
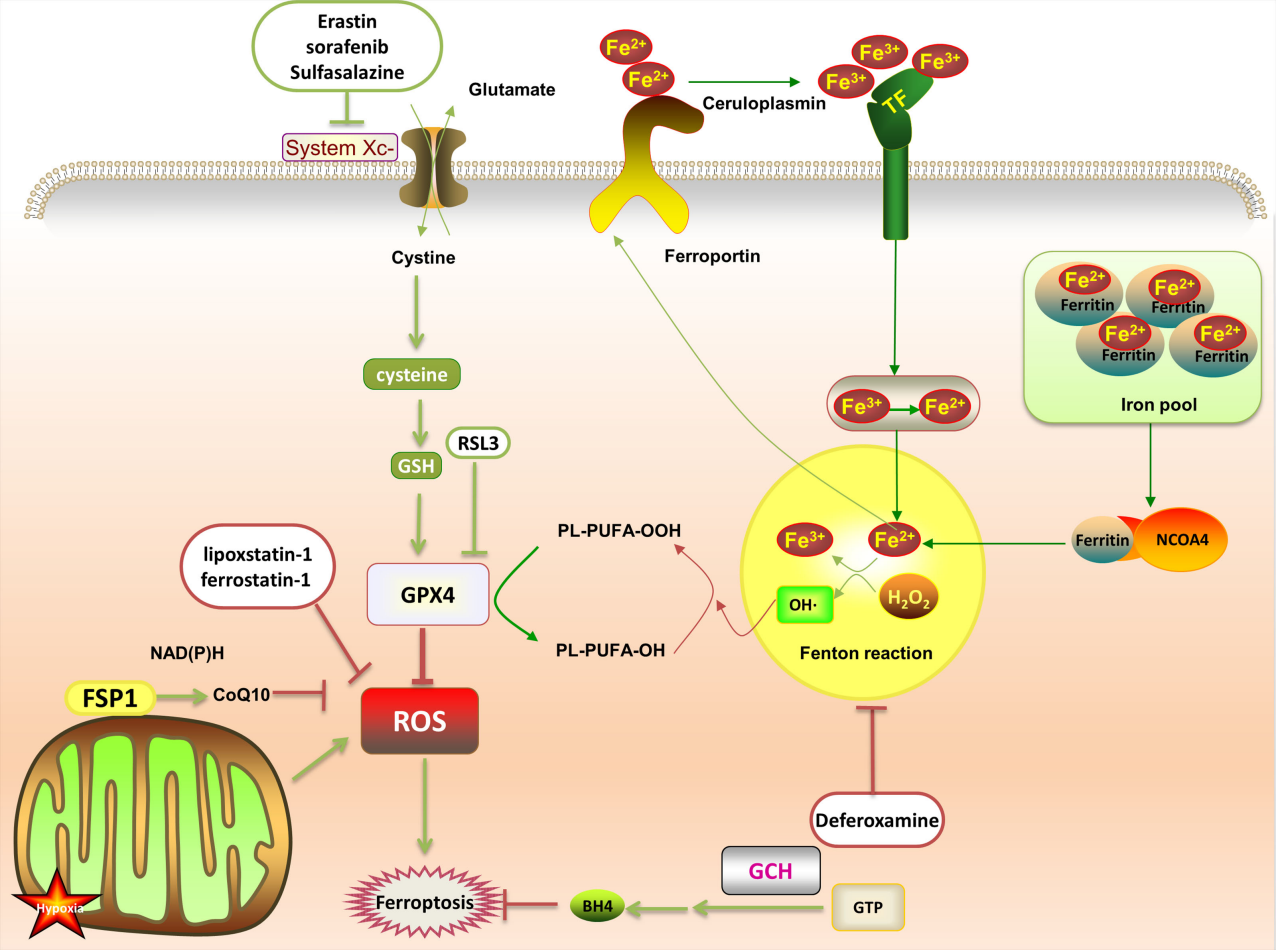
After the transferrin binds to the transferrin receptor on the plasma membrane, the plasma membrane forms a vesicle that takes Fe^3+^ carrying transferrin into the cell. Then, the low pH in the vesicle promotes the separation of Fe^3+^ from the transferrin and the shedding of Fe^3+^. It is reduced to Fe^2+^ and free in the cytoplasm, or is combined with ferritin to form an iron pool. The ferritin in the iron pool can be encapsulated by autophagy lysosomes under the mediation of NCOA4, and then degraded and release a large amount of Fe^2+^. Fe^2+^ and H_2_O_2_ generate PLOOH through the fenton reaction, which promotes ferroptosis by promoting further lipid peroxidation and self-peroxidation. In the GSH/GPX4 pathway, with the help of GSH, GPX4 down-regulates ROS and inhibits ferroptosis. This can be suppressed by RSL3. System Xc- (cystine/glutamate antiporter) promotes synthesis of glutathione, which can be offset by erastin, sulfasalazine and sorafenib. In the FSP1 protection pathway, FSP1 can catalyze the reduction of CoQ10 to panthenol and consume NAD(P)H to inhibit ROS. In the GCH1 protection pathway, GCH1 acts as a rate-limiting enzyme to manage the biosynthesis of BH4 and reduce ferroptosis.

Abnormal iron metabolism is an important cause of ferroptosis. Regulatory pathways of intracellular iron homeostasis mainly include ferroportin and TFR1 to regulate iron export and absorption (23). Iron ions induce the body to produce a large amount of lipid ROS through fenton reaction, promote lipid peroxidation, and lead to ferroptosis. Under oxidative stress conditions, superoxide will be produced in a short time, reducing Fe^3+^ stored in ferritin to Fe^2+^, resulting in the release of iron ions. Fe^3+^ enters the cell under the transport of TFR1, and then is converted into Fe^2+^. The excess iron ions are stored in ferritin to control the storage of iron ions. In addition, both iron response element binding protein 2 (IREB2) and Nrf2 are involved in regulating Fe^2 +^ in cells. NCOA4 recognizes and relies on the autophagy pathway to degrade intracellular ferritin, releasing free iron ions (28) (**Figure 1**).

The proliferation and activation of osteoclasts lead to RA bone damage and bone metabolism disorders. *In vitro* and *in vivo* studies have shown that iron overload can induce osteoclast differentiation and inhibit osteoblast proliferation by increasing ROS generation (29). Low concentrations of iron ions can promote the growth of osteoblast precursor cells (MC3T3-E1), while high concentrations of iron ions inhibit their growth and increase ROS levels (30). Iron overload can inhibit the activity of osteoblasts to a certain extent, thereby affect their differentiation process. Simultaneously, it can also activate the differentiation of osteoclasts and cause bone destruction (31). Studies have shown that excessive iron ions can activate p38-MAPK and block PI3K/AKT and JAK/STAT3 signaling pathways to induce MC3T3-E1 cell death (30). In addition, iron ions initiate the growth of synovial pannus by regulating the expression of critical genes such as *c-myc* and *mdm2*. These genes are responsible for the proliferation of synovial cells and promote the occurrence and development of vascular synovitis (32).

Studies have demonstrated that iron deposits are found in osteoarthritis and RA (33, 34). Both osteoarthritis and rheumatoid synovia contained iron, but in the latter greater quantities were present. However, none of the controls with normal synovia had iron deposition. Another study found that the iron metabolism is different in RA than in general health (35). It is worth noting that iron deficiency is common (64%) in RA patients with high disease activity. RA patients had lower hepcidin, lower transferrin, and lower ferritin. Icariin has antibacterial, anti-inflammatory and antioxidant effects (36). It has been suggested that icariin counteracts the effects of RSL3 on iron content, lipid peroxidation, and relative protein (SLC7A11, SLC3A2L, GPX4, TRF, NCOA4, and Nrf2) in synoviocytes given the observation that icariin might play a role in protecting synovial cells from ferroptosis (17). Herein, it can be exploited as a new therapeutic strategy for RA.

## Membrane Lipid Antioxidant System

GSH is a tripeptide containing sulfhydryl groups combined with glutamic acid, cysteine, and glycine, which has an antioxidant effect. Under normal circumstances, the cystine entering the cell is reduced to cysteine ​​to participate in the synthesis of GSH, which helps reduce the accumulation of lipid peroxides. However, when system Xc- is inhibited, GSH synthesis disorder will promote the decline of cellular antioxidant capacity and the accumulation of lipid ROS. Abnormally elevated lipid ROS levels can be controlled by GPX4 (37). GPX4 can effectively repair the oxidative damage of unsaturated fatty acids in mammals, thereby inhibiting ferroptosis. Stockwell et al. determined that the GPX4/GSH axis and the system Xc- regulate ferroptosis, considered a classic pathway (38). Earlier studies have shown that erastin, a system Xc- inhibitor, inhibits GSH synthesis and increases lipid ROS, leading to ferroptosis (21). Erastin has been shown to contribute to cartilage tissue damage by promoting matrix metalloproteinase 13 (MMP-13) expression and inhibiting type II collagen expression in chondrocytes (39), which may aggravate RA.

In addition to GPX4/GSH axis, ferroptosis suppressor protein 1 (FSP1)- Coenzyme Q10 (CoQ10) pathway has also been found to be related to ferroptosis. GPX4 and FSP1 are two parallel membrane lipid antioxidant pathways. FSP1 is one of the CoQ10 oxidoreductases, most of which are attached to the outer mitochondrial membrane (40). The FSP1-CoQ10 pathway can improve lipid peroxidation through free radical capture, and the process of ferroptosis is also blocked. Some studies speculate that FSP1 may improve RA through the TNF-α/ROS positive feedback loop (41, 42). However, the specific contribution of ferroptosis as a mode of cell death was not addressed in these studies.

It is worth noting that the GTP cyclohydrolase-1 (GCH1)-tetrahydrobiopterin (BH4) pathway is parallel but independent of the GPX4 and FSP1 pathways (43). GCH1 is the primary rate-limiting enzyme for the synthesis of BH4. Overexpression of GCH1 can enhance the production of BH4, and then reduce ferroptosis. So far, few such evidence has been provided for RA. Although the GCH1-BH4 protective pathway is closely related to ferroptosis, the interaction between GCH1-BH4 and RA is still not fully understood, and further research is needed.

## Oxidative Stress and Lipid Peroxidation

**Figure 2:**
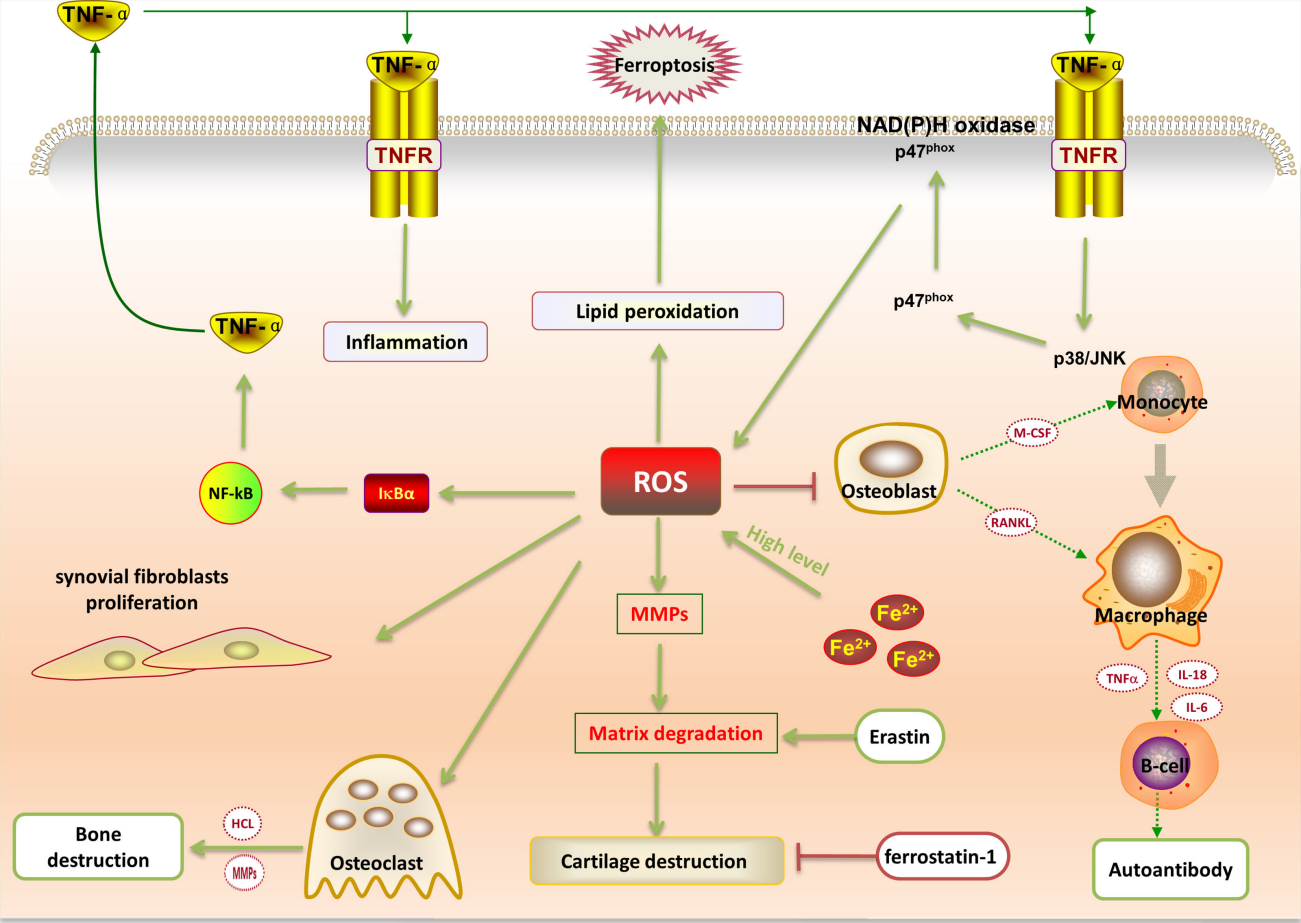
ROS is a critical element of the ROS/TNF-α feedback loop. The production of TNF-α depends on the activation of ROS-stimulated NF-κB signaling, which activates the p38/JNK signaling pathway to accelerate the progression of RA inflammation. High levels of iron ions can catalyze the production of ROS. Excessive ROS will aggravate the proliferation of synovial fibroblasts; induce osteoclast differentiation and inhibit osteoblast proliferation; activate metalloproteinases, as well as lead to cartilage destruction and bone erosion. Excessive ROS will also promote lipid peroxidation, leading to cell ferroptosis. Moreover, the ferroptosis inducer (erastin) can promote the expression of matrix metalloproteinase 13 and promote cartilage destruction, while the ferroptosis inhibitor ferrostatin-1 can reduce cartilage degradation.

Studies have shown that oxidative stress plays a vital role in the progression of RA. ROS, as a product of oxidative stress, exists in the articular cavity of RA patients in large quantities. ROS can be used as a potential marker for the progression of RA patients (44). Excess ROS can be converted to hydrogen peroxide through the fenton reaction. In this process, Fe^3+^ can be reduced to Fe^2+^, generating hydroxyl (-OH) or alkoxy (RO-) free radicals, and causing cell ballooning/blistering followed by plasma membrane rupture (45). ROS in cells can activate the NLRP3 receptor protein. Activated NLRP3 is polymerized by ATP to form highly ordered NLRP3 protein oligomers. Under the action of ASC, NLRP3 and pro-caspase-1 are connected to form a complex pro-caspase-1 that can be activated to form an enzymatic activity. The heterodimer caspase-1 cuts the inactive pro-IL-1β and pro-IL-18 into mature IL-1β and IL-18, aggravating RA. When the local inflammation of RA joints accelerates, it can be used as an endogenous signal regulator to expand the synovial inflammation response (46). ROS is a key element of the ROS/TNF-α feedback loop. The production of TNF-α depends on the activation of NF-κB signaling pathway stimulated by ROS, which in turn activates the p38/JNK signaling pathway to accelerate the progression of RA (41) (**Figure 2**). ROS can induce activation of metalloproteinases, inhibit the synthesis of cartilage proteoglycans, and promote chondrocyte apoptosis, which eventually leads to cartilage destruction and bone erosion. This is consistent with pathogenic manifestations of RA. A study detected a strong positive correlation between the ROS level and the severity score in RA patients (47). The levels of lipid peroxidation in the serum and synovial fluid are increased in RA patients, and the antioxidant system has also changed (48). Hence, ROS production in excess is more likely to inhibit osteoblast differentiation and lead to bone destruction.

In addition, studies have found that ROS production can also be induced by activation of the phagocyte NADPH oxidase 2 (NOX2) complex in a process generally referred to as an oxidative burst. NOX2-derived ROS have been shown to suppress antigen-dependent T-cell reactivity and remarkably to reduce the severity of experimental arthritis in both rats and mice (49). In addition, NOX2 also plays a role in antigen presentation and regulation of adaptive immunity. In CD4^+^ T cells, the lack of NOX2 induces the production of Th17 cells and reduces regulatory T cells in a ROS-dependent manner by affecting Foxp3 and RORγt (50). The immunosuppressive properties of CD4^+^CD25^+^Foxp3^+^Treg cells play a vital role in maintaining the body’s immune tolerance and immune response homeostasis. Early studies have demonstrated that regulatory T cells are functionally compromised in RA (51). The CD4^+^CD25^+^Treg cells in the joint synovial fluid of RA patients are significantly increased (52). In addition, the reduction of NOX2 will increase Th1, Th2, and Th17 cells, leading to inflammatory arthritis. Antigen-presenting cells (APCs) are known to produce NOX2-derived ROS. A study found that the NOX2-dependent processing of the redox-sensitive autoantigens by APCs modified T cell activity and induced development of RA in mouse models (53). Despite many unknown facts, drugs targeting ferroptosis may represent a potential strategy for treating RA.

## Inflammation

Ferroptosis can also trigger the body’s innate immunity, release inflammatory mediators, and activate the body’s inflammatory response (14). Changes in the synovial membrane’s typical physiological and metabolic properties can produce many inflammatory mediators, such as IL-1β, TNF-α, and IL-6, which increase uptake of transferrin and non-transferrin-bound iron by monocytes and increase the uptake of transferrin-bound iron by synovial fibroblast (54). Increased iron intake accelerates the vicious cycle of hemorrhage-synovitis-hemorrhage, and the proliferated synovial tissue spreads to the surface of the articular cartilage. Cartilage matrix is degraded by connective cathepsin released by hypertrophic synovial tissue, chondrocytes and intravascular tissue, which will eventually lead to the destruction of articular cartilage and bone (55, 56).

## Ferroptosis: A Rising Star With Great Therapeutic Potential in RA

P53 is an excellent tumor suppressor gene that can inhibit system Xc- uptake of cystine by down-regulating the expression of SLC7A11 and result in a decrease in antioxidant capacity. Previous studies have confirmed that p53 protein is expressed in RA fibroblast-like synovial cells, and its overexpression is a characteristic of RA (57). It plays a role in controlling the balance between Th17 cells and Treg (58). Aberrant p53/p21 activation-mediated aging-related secretory phenotype can accelerate destruction of cartilage tissue (59). Studies have shown that endogenous p53, which is inducible in rheumatoid synovial cells, is functionally active based on the findings that its expression blocks the G1/S transition by inhibiting the CDK-mediated phosphorylation of Rb *via* p21 induction (60). Clinical studies have found that the expression of p53 in lymphocytes is lower in RA patients than that of healthy people (61, 62). Acyl−CoA synthetase long−chain family member 4 (ACSL4, an enzyme involved in the activation of PUFAs) is located in peroxisomes and mitochondria, which can determine the sensitivity of cells to ferroptosis activation. Doll et al. found GPX4-ACSL double-knockout cells showed marked resistance to ferroptosis (63). Mechanistically, ACSL4 enriched cellular membranes with long polyunsaturated ω6 fatty acids. ACSL4 has a marked preference for activating PUFAs (64), therefore, deletion of ACSL4 prevents PUFAs from being incorporated into membrane PLs where they would become oxidized following GPX4 inactivation (26). Clinical studies have found that the ACSL4 is down-regulated in RA patients (65). BECN1 is a crucial regulator of autophagy, which can promote ferroptosis by regulating the activity of the system Xc-. BECN1-dependent ferroptosis requires the formation of BECN1-SLC7A11 complex. Studies have found that autophagy of osteoblasts affects bone metabolism, and BECN1 may become a new target for the treatment of bone metabolism diseases (66). CoQ10, a fat-soluble antioxidant, is a crucial regulator of ferroptosis. Studies have shown that CoQ10 has anti-inflammatory effects on autoimmune diseases. Jhun et al. used CoQ10-encoded liposome/gold hybrid nanoparticles targeting STAT3/Th17 to slow RA’s progression (67). Although the FSP1-CoQ10 protective pathway is closely related to RA, the interaction between CoQ10 and other ferroptosis regulators is still not fully understood, and further research is needed.

In addition, many studies have focused on the relationship between oxidative stress metabolism and ferroptosis regulators. For example, Nrf2 and heme oxygenase-1 (HO-1) level can regulate ferroptosis (68). A study found that reduced levels of the Nrf2 factor can lead to RA (69). Targeted activation of Nrf2 can inhibit ROS production, which in turn inhibits the proliferation and migration of RA fibroblast-like synovial cells (70). Luo H et al. found that RSL3 can reduce Nrf2 and GPX4 in synovial cells (17). In addition, lack of Nrf2 can lead to changes in the expression of SLC7A11, which further leads to oxidative stress damage and aggravates joint destruction (71). Studies have found that ferroptosis can be induced through the Nrf2-SLC7A11-HO-1 pathway, which may play a regulatory role in joint destruction (71, 72).

The study found that FDA-approved RA drugs such as sulfasalazine and auranofin can prevent cell growth and induce ferroptosis. Sulfasalazine and auranofin activity were largely mitigated by the ferroptosis inhibitor ferrostatin-1, antioxidants, or by the iron scavenger deferoxamine (DFO). DFO can inhibit ferroptosis by preventing iron ions from supplying electrons to oxygen to form ROS. However, the specific mechanism is still unclear. Dixon et al. made synthetic ferrostatin-1 (ferroptosis inhibitor) and proved that it could specifically inhibit ferroptosis, but it did not impede other oxidative substances and apoptosis-induced death (21). Yao et al. found that intra-articular injection of ferrostatin-1 increased the expression of collagen II, promoted the activation of the Nrf2 antioxidant system, and reduced cartilage degradation, which is beneficial to alleviate joint inflammation (39). Some natural polyphenol compounds can also significantly inhibit ferroptosis, such as baicalein, curcumin, and gastrodin (73, 74). Baicalein was demonstrated to suppress T cell proliferation in collagen−induced arthritis model mice and significantly improve T cell-mediated autoimmune diseases (75). Studies have confirmed that curcumin alleviates inflammation, synovial hyperplasia, and the other main features involved in the pathogenesis of collagen-induced arthritis (76). Targeting ferroptosis regulators may be a new direction for developing therapeutic drugs for RA.

## Discussion

In summary, ferroptosis is a recently discovered significant regulatory cell death pattern, and three protective pathways have been successively confirmed. An in-depth study of the underlying mechanism of ferroptosis is of great significance for mapping its role in various related autoimmune diseases. It is worth noting that different cell types (synovial cells, chondrocytes, osteoclasts, and macrophages) may have different susceptibility to ferroptosis. The expression profile of specific genes related to the ferroptosis pathway may not be observed in all cells. Moreover, attention should be paid to the phenomenon of ferroptosis, and the judgment criteria may vary depending on the trigger mechanism. Herein, it is necessary to study the mechanism of ferroptosis regulators. At present, the inner link between ferroptosis and RA has not been studied in depth. With an in-depth understanding of the relationship between ferroptosis and other biological processes, people will find that ferroptosis, apoptosis, autophagy, and other cell death patterns have some common characteristics in their regulations. Although simultaneous regulation of multiple cell death pathways is vital for the treatment of RA, the relationship between these different types of cell death is not yet fully elucidated. This requires further exploration to validate whether they are integrated into a complex regulatory network.

In short, with the unprecedented prosperity of research on ferroptosis, ferroptosis regulation represents a potential future avenue of investigation in the effort to identify novel therapeutic targets for RA.

## Author Contributions

TZ wrote the manuscript. DQ revised the manuscript. ZFL raised the idea for the article. YX, ZX, QY, JS, and ZML performed the literature search and data analysis. All authors contributed to the article and approved the submitted version.

## Funding

National Natural Science Foundation of China (31960178, 81960863, 81960870); Construction Project of National Traditional Chinese Medicine Clinical Research Base (2018 No. 131); Yunnan Provincial Fund for Medical Research Center: Clinical Evaluation and Basic Research on the Treatment of rheumatoid arthritis and gout by Traditional Chinese medicine (202102AA310006); Clinical Trial for the Treatment of Rheumatoid Arthritis with Warming yang and Smoothening Meridians (201507001-07, registration number: ChiCTR-INR-16010290); Clinical Cooperative Project of Chinese and Western Medicine for Major and Knotty Diseases; Yunnan Provincial Key Laboratory Construction Project Funding; Yunnan Provincial Key Laboratory of Chinese Medicine Rheumatology and Immunology; Yunnan Provincial “Ten Thousands Program” Famous Doctor Special; Yunnan Province Qingguo Wang Expert Workstation Construction Project (202005AF150017); Yunnan Applied Basic Research Projects-Union Foundation (2019FF002(-031)); Applied Basic Research Programs of Science and Technology Commission Foundation of Yunnan Province (2019FA007); Scientific Research Fund Project of Yunnan Provincial Department of Education (2021Y461).

## Conflict of Interest

The authors declare that the research was conducted in the absence of any commercial or financial relationships that could be construed as a potential conflict of interest.

## Publisher’s Note

All claims expressed in this article are solely those of the authors and do not necessarily represent those of their affiliated organizations, or those of the publisher, the editors and the reviewers. Any product that may be evaluated in this article, or claim that may be made by its manufacturer, is not guaranteed or endorsed by the publisher.
